# Two Novel Parvoviruses in Frugivorous New and Old World Bats

**DOI:** 10.1371/journal.pone.0029140

**Published:** 2011-12-27

**Authors:** Marta Canuti, Anna Maria Eis-Huebinger, Martin Deijs, Michel de Vries, Jan Felix Drexler, Samuel K. Oppong, Marcel A. Müller, Stefan M. Klose, Nele Wellinghausen, Veronika M. Cottontail, Elisabeth K. V. Kalko, Christian Drosten, Lia van der Hoek

**Affiliations:** 1 Laboratory of Experimental Virology, Department of Medical Microbiology, Center for Infection and Immunity (CINIMA), Academic Medical Centre (AMC), University of Amsterdam, Amsterdam, The Netherlands; 2 Institute of Virology, University of Bonn Medical Centre, Bonn, Germany; 3 Department of Wildlife and Range Management, Kwame Nkrumah University of Science and Technology, Kumasi, Ghana; 4 Institute of Experimental Ecology, University of Ulm, Ulm, Germany; 5 Gaertner & Collegues Laboratory, Ravensburg, Germany; 6 Institute of Medical Microbiology and Hygiene, University of Ulm, Ulm, Germany; 7 Smithsonian Tropical Research Institute, Balboa, Panama; Institut Pasteur, France

## Abstract

Bats, a globally distributed group of mammals with high ecological importance, are increasingly recognized as natural reservoir hosts for viral agents of significance to human and animal health. In the present study, we evaluated pools of blood samples obtained from two phylogenetically distant bat families, in particular from flying foxes (Pteropodidae), *Eidolon helvum* in West Africa, and from two species of New World leaf-nosed fruit bats (Phyllostomidae), *Artibeus jamaicensis* and *Artibeus lituratus* in Central America. A sequence-independent virus discovery technique (VIDISCA) was used in combination with high throughput sequencing to detect two novel parvoviruses: a PARV4-like virus named Eh-BtPV-1 in *Eidolon helvum* from Ghana and the first member of a putative new genus in *Artibeus jamaicensis* from Panama (Aj-BtPV-1). Those viruses were circulating in the corresponding bat colony at rates of 7–8%. Aj-BtPV-1 was also found in *Artibeus lituratus* (5.5%). Both viruses were detected in the blood of infected animals at high concentrations: up to 10E8 and to 10E10 copies/ml for Aj-BtPV-1 and Eh-BtPV-1 respectively. Eh-BtPV-1 was additionally detected in all organs collected from bats (brain, lungs, liver, spleen, kidneys and intestine) and spleen and kidneys were identified as the most likely sites where viral replication takes place. Our study shows that bat parvoviruses share common ancestors with known parvoviruses of humans and livestock. We also provide evidence that a variety of *Parvovirinae* are able to cause active infection in bats and that they are widely distributed in these animals with different geographic origin, ecologies and climatic ranges.

## Introduction

Bats (order Chiroptera, 1232 species [1]), the only mammals capable of actively sustained flight, are among the most diverse and species-rich vertebrate taxa. They play key roles in various ecosystems throughout the world [2], [3]. Furthermore, they also harbour pathogens and act as reservoir hosts of viruses that might be of relevance to human and domestic animal health [2]. Although the transmission of viruses from bats to humans has been proven only for rabies and some other lyssaviruses [3], as well as for Nipah [4] and Hendra virus [5] and assumed for SARS-CoV-like viruses [6], a wide range of highly pathogenic viruses have been detected in bats, e.g. Ebola [7] and Marburg [8] viruses. As a consequence, virus discovery techniques have in recent years been used widely to identify previously unknown viruses in bats and have led to recognition of numerous new species in saliva, faeces as well as in respiratory and alimentary specimens (e.g. astroviruses [9], coronaviruses [10], paramyxoviruses [11]. In some cases the identified viruses (e.g. coronaviruses) are phylogenetically related to human viruses [10] indicating possible prior transmission to humans, although for most viruses there are no indications for recent zoonotic transmissions from bats to humans. The majority of the newly described bat viral species are RNA viruses, but several DNA viruses (e.g. circoviruses [12], polyomaviruses [13], adenoviruses [14] and herpesviruses [15]) have also been detected recently. In addition, two studies describe the identification of members of the *Parvoviridae* family in fecal samples of different species of bats [12], [16]

The *Parvoviridae*, comprising the subfamily *Parvovirinae* (viruses that infect vertebrates) and the subfamily *Densovirinae* (viruses that infect arthropods), are non-enveloped viruses, containing a single stranded DNA genome of approximately 5 kb. The *Parvovirinae* subfamily is currently subdivided into 5 well established genera (*Parvovirus*, *Amdovirus*, *Erythrovirus*, *Dependovirus*, *Bocavirus*) and a new genus (PARV-4 like viruses) that contains 5 viruses that have recently been identified: Human Parvovirus 4 (PARV4, identified in humans in 2005) [17] classified into three genotypes, Bovine Hokovirus (BoHV, 2008) [18], Porcine Hokovirus (PoHV, 2008) [18] [19], and a Chimpanzee and a Baboon PARV4-like virus (2010) [20]. Some of the *Parvovirinae* viruses require a co-infection with a helper virus to be perpetuated (the majority of the viruses within the genus *Dependovirus*), but most can replicate autonomously.

In the present study we evaluated whether bats carry viruses that belong to the *Parvovirinae* subfamily, in particular viruses that are closely related to the human ones in search for ancestral viruses and new potentially zoonotic viruses. So far virus discovery in bats was predominately performed in stool samples [9], [12], [14], [16], but we chose serum/EDTA-plasma samples since human parvoviruses can appear in high concentrations in blood [21]. Three bat species were examined: *Eidolon helvum* and *Artibeus jamaicensis/A. lituratus*, frugivorous bats from Ghana and Panama, respectively. A sequence independent virus discovery technique (VIDISCA) was used in combination with high throughput sequencing [22]. By applying this methodology two new parvoviruses were identified. To further analyze the characteristics of these new viruses, the nearly full-length genome sequences were determined, their prevalence in the respective populations was investigated, and viral concentrations in blood and different organs were determined.

## Materials and Methods

### Bat sampling

Serum and organ samples were collected from wild *Eidolon helvum* (straw-coloured fruit bats, estimated colony size 300,000) in Kumasi, Ashantia Region, Ghana, in 2009. For all capturing, sampling and exports permission was obtained from the Wildlife Division of the Ministry of Lands, Forestry, and Mines in Ghana (Permit A04957 of 28 April 2009, Research Project Code 03/04/2009, granted to S. Oppong). Ethics approval was given by the Kwame Nkrumah University of Science and Technology (KNUST Committee on Human Research, Publications and Ethics, CHRPE49/09, granted to Y. Adu-Sarkodie). Research samples were exported under a state agreement between the Republic of Ghana and the Federal Republic of Germany, represented by the City of Hamburg. Additional export permission was obtained from the Veterinary Services of the Ghana Ministry of Food and Agriculture. Whole EDTA blood samples from Panamanian *Artibeus jamaicensis* and *A. lituratus* fruit bats were collected during an ecological study on haemoparasites (November 2005) in a tropical lowland forest (Barro Colorado Nature Monument) in Panama [23], [24] with respective permits for field work and export obtained from the Smithsonain Tropical Research Institute (STRI; IACUC protocol approved for V. Cottontail) and the Panamanian Autoridad Nacional del Ambiente (ANAM; Export permit # SEX/A-145-05 granted to V. Cottontail).

### VIDISCA-454

Samples for virus discovery were tested in pools: 10 pools each containing 2 serum samples collected from Ghanaian bats, one pool containing 50 EDTA-plasma samples derived from Panamanian bats.

VIDISCA-454 (virus discovery cDNA-AFLP, Amplified Fragment–Length Polymorphism combined with Roche 454 high-throughput sequencing) was performed with 110 µl of pooled serum/EDTA-plasma samples as previously described [22].

### Sequence analysis and nearly full-length genome sequencing

Primer and MID (Multiplex Identifier) sequences were trimmed from every read. Sequences were then assembled with CodonCode Aligner software version 3.5.6. Contigs and unassembled sequences were compared to known nucleotide and protein sequences contained in GenBank database using different BLAST tools (blastn, blastx and tblastx) [25].

Identified viral fragments were used as a template for primer design (sequences available on request) to obtain the nearly complete genome sequences. Sequencing was performed employing Big Dye terminator chemistry (BigDye® Terminator v1.1 Cycle Sequencing Kit, Applied Biosystems, Nieuwerkerk aan de IJssel, The Netherlands).

### Molecular screening and parvovirus quantification in blood and organs

DNA from 100 serum samples collected from Ghanaian *Eidolon helvum* bats was isolated by the QIAamp MinElute Virus Spin Kit (Qiagen, Hilden, Germany) according to the manufacturer's recommendations. The input volume was 200 µl, the elution volume was 120 µl. Up to 200 µl of whole EDTA blood collected from 92 Panamanian *Artibeus jamaicensis* bats and from 18 Panamanian *Artibeus lituratus* bats were mixed with 500 µl lysis buffer (10 mM Tris-HCl pH 8.0 100 mM EDTA, 2% SDS) and DNA was prepared by using the QIAamp Blood DNA Mini Kit (Qiagen, Hilden, Germany) according to the manufacturer's instructions except for an initial 15 sec incubation with proteinase K [23]. Eluate pools were then created as follows: 10 pools (10 eluates each) were prepared with 40 µl of each DNA isolate from Ghanaian *Eidolon helvum* serum samples and 22 pools (5 eluates each) were made with 5 µl of each DNA preparation from Panamanian *Artibeus* bats specimens.

To test the organ distribution of the virus and to detect the potential replication site, DNA was also isolated from organ tissues of *Eidolon helvum* bats found to be parvovirus-positive. Samples collected from brain (n = 7), lungs (n = 7), liver (n = 6), kidneys (n = 7), spleen (n = 7), small intestine (n = 5), and large intestine (n = 4) were analyzed. Nucleic acid was prepared from 20 mg of tissue by using the TissueLyser method with steel beads (Qiagen, Hilden, Germany) and proteinase K in ATL buffer followed by the QIAamp DNA Blood Mini Kit procedure (Qiagen, Hilden, Germany). DNA was eluted in 100 µl.

DNA preparations from single samples and from pools were tested for the presence of the newly identified viruses through homemade hemi-nested or nested PCRs (see table 1 for primer sequences).

**Table 1:** Primers used in this study for screening, sequencing and quantifying Eh-BtPV-1 and Aj-BtPV-1.

*Eidolon Helvum*	
Screening and Sequencing	
EhParvo-fouter	TCCTTGTCCCCGGCTACAATTATGT
EhParvo-reverse	GGAGGATTAGGGGGCAAAACCTG
EhParvo-finner	GTGGGTCCTGGTAATCCTTTGGAT
**Quantification**	
EhParvo-quant-forward	GAGGCAGCTCGCAATCATG
EhParvo-quant-reverse	GACGTCCCCGTGGGAAA
EhParvo-probe	FAM-CAGAAGGTATGACGAAATG-MGBNFQ

*Artibeus*	
Screening and Sequencing	
AjParvo-fouter	TGCACAACCCACCACCTCAGATA
AjParvo-reverse	CCACCACCTCAGATACTGCTAAAA
AjParvo-finner	ACACGGAATCGGGTAGTCCAC
jParvo-N-fouter	GGACTTTGCATAATGGAAGACACAG
AjParvo-N-router	GTGGCGTATTGTTTCARGTAGGA
AjParvo-N-finner	GTATGGGACACACGATCGCTGTAYTA
AjParvo-N-rinner	GTTGTGGTGTTATATGCTGGCAT
**Quantification**	
AjParvo-quant-forward	GTWTACCCCATGCCAGCATAT
AjParvo-quant-reverse	CTTGGTTTTACGTCCCATGTCA
AjParvo-probe	FAM-CAGCWGGAAAATCCTACYTGAAACAATACG-BHQ1

Amplicons from the hemi-nested PCRs were TA cloned into the pCR®4-TOPO® plasmid vector according to the instructions of the manufacturer (Invitrogen, Karlsruhe, Germany). Plasmid DNA was prepared by using the QIAprep Spin Miniprep Kit (Qiagen, Hilden, Germany). Nucleic acid concentration was determined spectrophotometrically.

Absolute quantification of viral DNA (copies/ml of serum or whole blood, copies/g of tissue) was achieved by real-time qPCR using plasmid-based standards and primers and probes listed in table 1 (HotstarTaq Plus Master Mix Kit, Qiagen, Hilden, Germany).

### Phylogenetic analysis and genome characterization

Fifty-nine reference nucleotide sequences and amino acid sequences were downloaded from the GenBank database and aligned together with the ones obtained in this study using ClustalX software version 2.0.12 [26] and the Cobalt Multiple Alignment Tool (http://www.ncbi.nlm.nih.gov/tools/cobalt/) for nucleotide and amino acidic sequences, respectively. Only complete coding sequences were included in the analysis. Alignments were manually edited when needed with BioEdit software version 7.0.5.3 [27] and then used for phylogenetic inference. The evolutionary distances were computed using the Maximum Composite Likelihood method for nucleotides [28] and the Poisson Correction [29] for proteins. Phylogenetic trees were constructed by means of MEGA software version 4.0.2 [30] using the Neighbor-Joining method [31]. To test the robustness of the analysis a bootstrap test (1000 replicates) [32] was performed and only clusters associated with a value higher then 75% were considered significant. Identities between and within sequences were calculated with BioEdit software version 7.0.5.3 [27]: the positions where both sequences have a gap did not contribute and positions with a gap in only one sequence counted as mismatch.

To examine whether the viruses were recombinant viruses, Bootscanning analysis [33] (Kimura 2-Parameter model [34]; window size, 200 bp; step size, 20 bp; Gap strip: on; 1000 bootstrap replicates and neighbour-joining) were performed with Simplot software version 3.5.1 [35]. The same software (with the same settings) was used to perform similarity scans and thus identify conserved regions between viruses. Open Reading Frames (ORF) were detected by the NCBI ORF-Finder tool (http://www.ncbi.nlm.nih.gov/projects/gorf/).

### Accession Numbers

All sequences obtained in this study have been deposited in the GenBank database under accession numbers JQ037753–JQ037754 (reference strains) and JQ037755–JQ037761 (partial sequences).

## Results

### Identification of *Parvovirinae* in bats

VIDISCA is a virus discovery technique that can identify RNA and DNA viruses, without prior knowledge of its genome sequence. The technique was successfully used to discover human coronavirus NL63 [36]. Nowadays, VIDISCA is combined with second generation sequencing techniques and the method allows identification of viruses in clinical samples [22]. In search for previously unknown bat parvoviruses we combined VIDISCA with Roche-454 sequencing and generated high amounts of sequence reads from pools of bat blood derived from various bat species originating from Ghana and Panama (in total 227931 reads). In one pool of 2 serum samples collected from frugivorous *Eidelon helvum* bats from Ghana and in one pool of 50 EDTA blood samples collected from frugivorous *Artibeus jamaicensis* bats from Panama 136 parvoviral hits (0.65% of the *Eidelon helvum* bat reads, resulting in 15 different fragments) and 168 parvoviral hits (2.4% of the *Artibeus jamaicensis* bat reads, resulting in 22 different fragments) were identified. Respectively 42.5% and 52.5% of the complete genome were obtained with VIDISCA-454.

The identified viral fragments were used as template for primer design and with specific PCRs the original positive samples were detected. Via genome walking it was possible to amplify and sequence the viral genome from 2 previously unknown members of the *Parvovirinae* subfamily (5065 nt for the Ghanaian virus and 4595 nt for the Panamanian virus), although sequences of the terminal hairpins are missing.

No other reads with clear identity to any known viruses were detected in any of the samples.

### Ghanaian *Eidolon helvum* parvovirus

#### Genome organization and phylogenetic analysis

**Figure 1:**
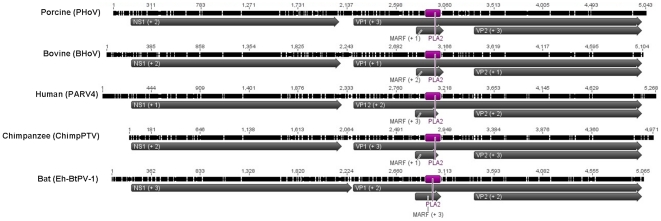
Genome organization of Eh-BtPV-1 and of representative members of the PARV4-like genus. Indicated are the main ORFs (gray, coding frame indicated in parenthesis) and the PLA_2_ motif position (purple). On the top the identity within the alignment is shown. Accession numbers: PHoV, EU200673; BHoV, EU200670; ChimpPTV, HQ113143; PARV4, AY622943. Figure was made with Geneious v5.1 software [52].

The genomic organization of the parvovirus detected in *Eidolon helvum* bats is similar to the other members of the *Parvovirinae*, with two large ORFs. The 5′ ORF (nucleotides 174–2252) putatively encodes for the non structural protein (NS1, 692 aa) and the 3′ ORF (nucleotides 2279–5038) for structural viral proteins VP1 (919 aa) and VP2 (544 aa). Moreover, by using ORF finder an additional ORF (Middle Alternative Reading Frame, MARF, nucleotides 2829–3062) and the corresponding putative protein (77 aa) were also identified (Fig. 1).

**Figure 2:**
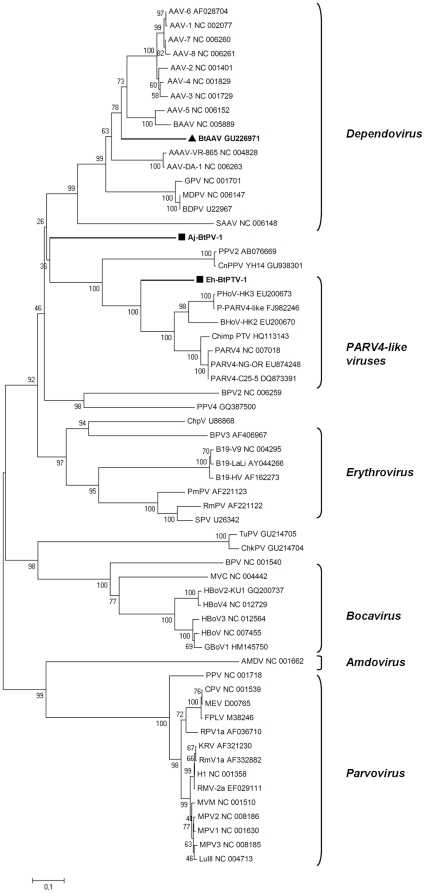
Phylogenetic relationship of bat parvoviruses with known *Parvovirinae*. NS1 protein phylogenetic analysis of Eh-BtPV-1 and Aj-BtPV-1 with 59 reference NS1 proteins obtained from GenBank. Genera as well as reference strains' accession numbers are indicated. Members of the *Parvovirinae* detected in bats are in boldface. The squares indicate the viruses described in this study and the triangle indicates a virus recently identified in bat faeces [16].

Phylogenetic analysis of the *Eidolon helvum* parvovirus was performed with full genome as well as with separate genes and proteins. In all the analyses the new bat virus clustered with the PARV4-like viruses (cluster supported by a bootstrap value of 100 on the NS1 protein tree shown in Fig. 2). We named the novel virus *Eidolon helvum* Parvovirus 1 (Eh-BtPV-1). Phylogenetically, Eh-BtPV-1 was located between the Porcine Parvovirus 2 (PPV2) and the PARV4-like groups resembling a separate branch near the root of the PARV4-like genus thus potentially sharing a common ancestor with human PARV4, the Porcine (PoHV), the Bovine (BoHV), and the Chimpanzee (ChimpPTV) PARV4-like viruses (Fig. 2).

To better clarify the position of Eh-BtPV-1 within the PARV4-like viruses, the molecular characteristics and identities, both at nucleotide and at amino acid level, between the species were considered. Comparing all the members of the genus, Eh-BtPV-1 has the largest NS1 protein (692 aa vs. 663 aa of the primate viruses, 652 aa of the bovine viruses and 636 aa of the porcine viruses). The NS1 protein of Eh-BtPV-1 is equally distant from NS1 of all the other PARV4-like viruses, being on average 41.4% amino acid identical to primates viruses, 42.4% identical to the bovine virus and 43.8% identical to porcine virus, and is thus the most dissimilar within the genus (identities within NS1 of all viruses excluding the one of Eh-BtPTV-1 ranges between 53.9% and 96.8%, Table 2).

**Table 2:** Identity ranges (%) of the 3 ORFs both at amino acid (bold) and nucleotide level between species in the PARV4-like viruses^a^.

	Eh-BtPV-1	PARV4-g1	PARV4-g2	PARV4-g3	ChimpPTV	PHoV	BHoV
**Eh-BtPV-1**							
ORF1	-	**41.4**	**41.1**	**41.3**	**41.8**	**43.8**	**42.4**
ORF2	-	**50.7**	**50.3**	**50.5**	**49.7**	**50.2**	**48.6**
MARF	-	**45.4**	**45.4**	**45.4**	**46.7**	**45.3**	**43.5**
**PARV4-g1**							
ORF1	48.5	-	**96.8**	**96.5**	**90.9**	**54**	**56.3**
ORF2	56.2	-	**98.6**	**98.4**	**93.2**	**65.4**	**65.4**
MARF	59.4	-	**100**	**100**	**97**	**48.2**	**47.6**
**PARV4-g2**							
ORF1	48.8	90.9	-	**97.1**	**91.5**	**54.3**	**56.5**
ORF2	55.1	93.4	-	**98**	**93.2**	**65.2**	**65.4**
MARF	58.9	99.5	-	**100**	**97**	**48.2**	**47.6**
**PARV4-g3**							
ORF1	48	89.6	89.2	-	**90.7**	**53.9**	**56.8**
ORF2	55.7	93.6	92.9	-	**92.9**	**65.1**	**64.9**
MARF	59.4	100	99.5	-	**97**	**48.2**	**47.6**
**ChimpPTV**							
ORF1	48.5	81.2	82	79.8	-	**54.6**	**56.3**
ORF2	54.9	85	84.7	84.5	-	**64.5**	**64.9**
MARF	59.8	98.5	98	98.5	-	**48.2**	**47.6**
**PHoV**							
ORF1	46.5	55.1	55.3	54.7	55.6	-	**67.7**
ORF2	53.1	62.9	62.9	63	63.4	-	**66.1**
MARF	60.1	59.3	59.3	59.3	59.3	-	**71.7**
**BHoV**							
ORF1	47.6	57.1	57.7	56.7	57.9	62.4	-
ORF2	52.6	63.8	63.5	63.4	63.3	64.2	-
MARF	61.2	60.3	60.3	60.3	60	79.4	-

a Eh-BtPV-1 (*Eidolon helvum Bat Parvovirus* 1), PARV4-g1/3 (*Human Parvovirus 4* genotypes 1, NC_007018, 2, DQ873391, 3, EU874248), ChimpPTV (*Chimpanzee PARV4*, HQ113143), PHoV (*Porcine Hokovirus*, EU200673), BHoV (*Bovine Hokovirus*, EU200670).

The VP1 protein of Eh-BtPV-1 (919 aa) coded by ORF2 appears to be larger than VP1 of the primate PARV4-like viruses (914 aa) but smaller than the one of the porcine hokovirus (925 aa) and the VP1 of the bovine virus (931 aa) (Fig. 1). Like NS1, VP1 protein of Eh-BtPTV-1 is similarly equidistant from the VP1 protein of other PARV4-like viruses, with 48.6% identity with VP1 protein of the bovine virus, 50.2% with the porcine virus and on average 50.3% with the primate and human viruses. Compared to NS1, Eh-BtPV-1 VP1 is more related to the other VP1 proteins in the genus but it is still the most diverse sequence (identities within VP1 of all viruses excluding the one of Eh-BtPV-1 ranges between 65.1% and 98.6%, Table 2).

The VP1 of Eh-BtPV-1 contains the phospholipase A_2_ motif, which is typically present in most of the parvoviruses but absent in other viral families. This motif (PLA_2_, aa 216–260 of VP1 protein) is located in the N-terminal VP1 region that does not overlap the VP2 protein (VP1 unique region). As expected, this motif was conserved among all PARV4-like viruses. The catalytic domain of PLA_2_ (HDXXY) had only one difference in Eh-BtPV-1 compared to all the other viruses (HDRRY vs. HDERY) whereas the sequence of the Ca^++^ binding loop (YXGXG) was identical in all the viruses (YVGPG).

By comparing the ORF patterns of all the PARV4-like viruses, we noticed that the genus is characterized by the presence of the aforementioned small open reading frame: MARF. This ORF overlaps with the PLA_2_ motif in the VP1 unique coding region. In all PARV4-like viruses the MARF is in frame +1 in respect to the VP1 gene (Fig. 1) but varying in size amongst the viruses (67 aa in primate viruses, 84 aa in both porcine and bovine viruses and 77 aa in Eh-BtPV-1).

#### Prevalence and tissue distribution

Out of 100 serum samples that were tested by hemi-nested PCR, 7 (7%) resulted positive for the presence of Eh-BtPV-1. Viral concentrations ranged from 2.84E+4 to 1.47E+10 copies/ml (Table 3).

**Table 3:** Detection of Eh-BtPV-1 in different body compartments of 7 *Eidolon helvum* bats.^a^

Bat	Serum	Liver	Spleen	Kidney	Lung	Brain	Small intestine	Large intestine
1	1.15E+08	1.81E+08	**5.00E+09**	**8.55E+10**	8.40E+08	2.60E+07	NA	NA
2	2.80E+06	1.34E+05	4.74E+04	1.29E+05	NEG	3.05E+04	NA	NA
3	2.84E+04	1.62E+06	**3.44E+10**	**1.18E+09**	2.52E+06	1.34E+05	**3.03E+07**	NA
4	1.37E+10	4.58E+09	**1.93E+11**	5.50E+09	1.27E+10	7.40E+08	2.91E+10	6.70E+09
5	1.47E+10	NA	7.02E+05	1.42E+06	2.36E+05	1.65E+04	9.75E+04	7.05E+04
6	4.15E+05	5.10E+05	**4.32E+07**	**5.00E+08**	9.20E+04	4.26E+04	1.46E+05	5.40E+04
7	4.91E+04	1.65E+04	**1.06E+07**	**3.01E+06**	8.25E+05	NEG	1.09E+05	6.60E+04

a Viral concentrations are indicated as copies/ml in serum and copies/g of tissue obtained from different organs; NEG = negative; NA = not available. The boldface viral concentrations indicate organs with a concentration at least 10 fold higher than serum.

Additionally, Eh-BtPV-1 was found in all types of tested organs (liver, spleen, kidney, lung, brain, small and large intestine) and, in most cases, at high titers (Table 3). Based on quantification assays, it may be possible to identify spleen and kidney as the potential replication sites of Eh-BtPV-1, because virus concentration found here was in most cases (71.4% of spleens and 57.1% of kidneys) at least 10 fold higher than in the blood compartment. Although a viral filtration activity could not be excluded for these organs since in two cases viral concentration was here lower than in blood.

The sequences obtained from the Eh-BtPV-1-positive bats (both from blood and organs) were 98.7–100% identical to each other, demonstrating the circulation of a single type in the analyzed bat population (data not shown). Beyond this, all the sequences obtained from samples belonging to the same bat (but different body districts) were identical, showing no apparent variability during the infection.

### Panamanian *Artibeus jamaicensis* parvovirus

#### Genome organization and phylogenetic analysis

Like Eh-BtPV-1, the genomic organization of the novel parvovirus identified in Panamanian *Artibeus jamaicensis* bats, exhibits characteristics common to the other vertebrate parvoviruses.

Phylogenetic analysis showed no clustering in the different trees and no clearly identifiable closest relative in any of the known genera (Fig. 2). In all the analyses the novel bat virus formed a separate branch giving indications that this is the first member of a new genus in the *Parvovirinae* subfamily. The virus was named *Artibeus jamaicensis* Bat Parvovirus 1, Aj-BtPV-1.

To identify conserved parts between *Parvovirinae* a Simplot analysis was performed. In the first putative ORF (nucleotides 84–1961 of Aj-BtPV-1, coding for a putative non structural protein NS1 of 625 aa) it was possible to identify a more conserved region of approx. 500 nucleotides. This region (nucleotides 835–1320 of Aj-BtPV-1 NS1 ORF) was then used to perform additional alignments and create further phylogenetic trees (both at nucleotide and amino acid level). The top identity percentages of Aj-BtPV-1 were found with *Dependoviruses* (between 53.4 and 59.6% at the amino acid level). However, all trees were consistent with the previous ones and Aj-BtPV-1 was still forming a separate lineage (branching autonomously from the others) confirming its independence from all the other known genera.

Since this virus represents the first member of a potentially new parvoviral genus and the transcriptional profile is not yet available, two alternative start codons could be hypothesized as the transcriptional origin of the VP1 protein. The first one (at position 1965 of the sequenced genome) is only 3 nucleotides after the stop codon of the first putative ORF and it would give origin to a protein of 876 aa (stop codon starts at nucleotide 4593); the second start codon is located at position 2025 and the product of this ORF would be a protein of 856 aa.

The PLA_2_ domain with the conserved catalytic site (HDRGY) and the Ca^++^ binding loop (YTGPG) was identified at amino acids 259–303 of VP1 protein.

#### Prevalence

**Figure 3:**
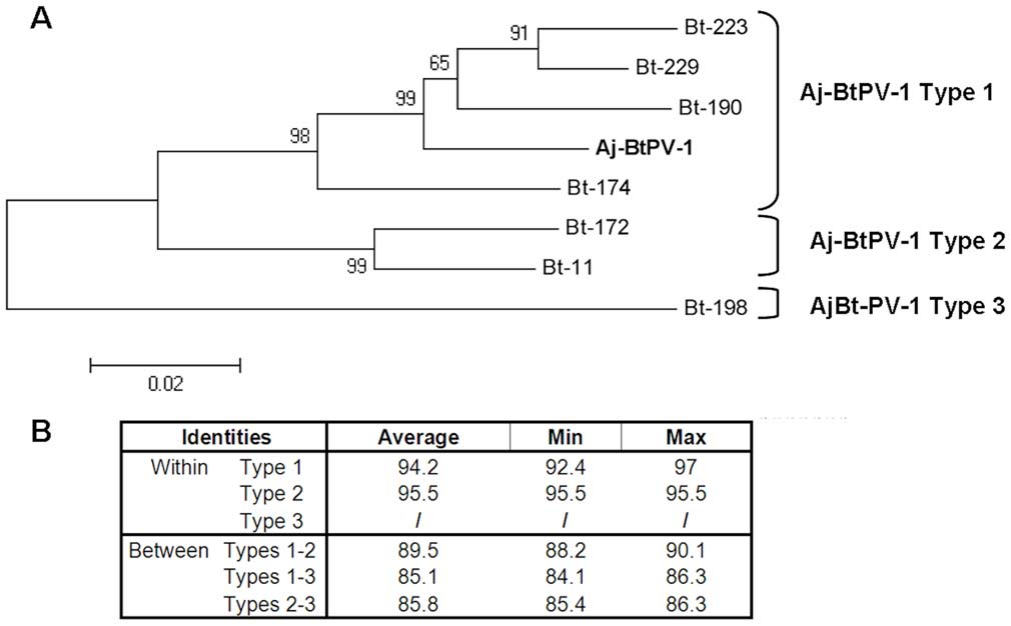
Three types of Aj-BtPV-1. The phylogenetic tree was based on a 740 nt fragment (nucleotides 3820–4561) of Aj-BtPV identified in *Artibeus jamaicensis* and *Artibeus lituratus* (#174) bats from Panama, showing the 3 different viral types (panel A). Identity values (in percentage) within and between the 3 types are shown in panel B.

Out of 110 whole EDTA blood samples, 8 bats (7.3%) tested positive for the presence of Aj-BtPV-1, with a viral concentration ranging from 8.3E+03 to 4.87E+08 copies/ml. Seven of the Aj-BtPV-1-positive animals belonged to the frugivorous bat species *Artibeus jamaicensis* (7/92, 7.6%) and one animal (#174 in figure 3) belonged to the closely related frugivorous bat species *Artibeus lituratus* (1/18, 5.5%).

Based on phylogenetic analysis of a 740 nt fragment (nucleotides 4329–4561) amplified from different bats, three Aj-BtPV-1 types were distinguishable (Fig. 3). Types 1 and 2 were the most closely related towards each other (mean between identity of 89.5%, average identity within the types of 95.2% and 95.5%), whereas type 3 (only one sequence available) was the most diverse one (mean between identity of 85.1% and 85.8% vs. types 1 and 2, respectively) (Fig. 3). This variability was also visible at the amino acid level: 28 out of 246 variable sites could be noted. The virus detected in the bat species *Artibeus lituratus* belonged to the type 1 cluster.

## Discussion

Globally, bats are, among mammals, one of the most diverse and species-rich groups of vertebrates and, together with humans and rodents, they are the most broadly dispersed mammalian species. They live frequently in large groups and can reach very high densities in roosts where up to millions of bats may cluster in tight spaces [2]. Bats are the only mammals able to fly and some species regularly travel more than 3000 km crossing large stretches of land and water barriers [37]. All these characteristics offer the opportunity to pathogens to easily be maintained within the population and to be diffused in other populations and different geographical areas. The scientific community increasingly recognizes bats as natural reservoir host for many zoonotic diseases including a wide range of viruses with important implications for human and animal health [5], [7], [38], [39].

Among the most important molecular characteristics that are likely to contribute to the emergence of a virus in a novel host system is the ‘evolutionary potential’: a rapid mutation rate and the tendency to recombine make a virus more prone to spread in a new host species [40]–[42]. Among DNA viruses, parvoviruses exhibit the highest mutation rate, almost comparable to that possessed by RNA viruses, and they are extremely recombination prone [43]. Furthermore, host-switching has been reported [40], although co-divergence (lead by co-evolution) of parvoviruses and their host is also noticed [44]. All these characteristics make parvoviruses one of the most likely candidates to be future emerging viruses. For these reasons it is of extreme importance to further pursue the search for novel parvoviruses and to clarify the real impact of the circulation of these viruses in bats and other mammals to identify and better characterize mechanisms linked to zoonotic transmission and host switching.

To evaluate whether bats carry viruses belonging to the *Parvovirinae* subfamily, we analyzed blood samples from two phylogenetically distant bat families [45] living in distant geographical areas. Both groups share similar ecological traits as they both feed on fruits as main diet. We used VIDISCA, a sequence independent virus discovery technique, combined with Roche-454 high throughput sequencing and discovered 2 novel members of the subfamily *Parvovirinae*: Eh-BtPV-1, a PARV4-like virus, identified in Ghanaian *Eidelon helvum* bats and Aj-BtPV-1, initially identified in Panamanian *Artibeus jamaicensis* bats and representing the first member of a putative new parvoviral genus.

To our knowledge, Eh-BtPV-1 is the first potentially autonomously replicating member of the subfamily *Parvovirinae* identified in bat blood. Eh-BtPV-1 exhibits monophyletic origin with human PARV4 and the other primate, bovine and porcine viruses more recently identified [17]–[20]. Unfortunately the baboon virus could not be included in the phylogenetic analysis due to the limited length of baboon viral sequence that is available (296 nts). Interestingly, based on computational prediction, beyond the putative major ORFs of Eh-BtPV-1 a small ORF termed MARF was additionally found in the unique part of VP1 overlapping the PLA_2_ motif, but in a different reading frame. MARF was subsequently detected in all PARV4-like viruses so far known suggesting that the presence of this ORF characterizes this putative genus. Besides, Eh-BtPV-1 resembles a separate branch within the genus and, amongst the PARV4-like viruses identified until now it is the virus closest to the root of the genus. These data may suggest that a possible zoonotic transmission could have occurred in the past allowing the ancestor virus to be transmitted from bats to other mammals, as it has been already proposed for SARS-CoV [6] and other coronaviruses [10]. Since Eh-BtPV-1 is equally divergent from primates, bovine and porcine viruses, an independent introduction of an ancestral bat virus into different mammalian species can be hypothesized. It can also be assumed that either humans or other mammals could have played a role in transmitting the ancestral virus from bats to the other species. However, for exactly clarifying the role of bats in the evolution of this group a greater spectrum of PARV4-like viruses potentially present in bats has to be elucidated.

*Eidolon helvum*, the most widely distributed fruit bat species in Africa, is a transcontinental migrant seasonally forming huge colonies of 5 to 10 million individuals. Its primary habitat is equatorial Africa but flying-foxes can migrate more than 2500 km [46]. In the investigated population, the prevalence of the Et-BtPV-1 was 7% and the virus could be detected in all tissues tested, with spleen and kidneys as most likely candidates for potential replication sites. Since the colonies are often in close contact with the human population and in some African areas the bats are habitually used as source of food [47], as is the case with *Eidolon helvum* in Ghana [48], studies are needed to investigate whether the virus could be transmitted to a human host, e.g., by antibody studies in individuals involved in bat bush meat processing.

The second virus identified in this study, Aj-BtPV-1, was initially found in *Artibeus jamaicensis* bats from Panama. Aj-BtPV-1 was circulating in the respective *A. jamaicensis* population at a rate of 7.6% and, based on phylogenetic analysis, it clearly appears that at least three different lineages are currently circulating in this bat species. Interestingly, Aj-BtPV-1 was also found in blood collected from a bat belonging to the closely related species *A. lituratus*. Although the number of analyzed *A. lituratus* bat is low (18), the prevalence of viraemia (5.5%) was close to that detected in *Artibeus jamaicensis* bats. Aj-BtPV-1 is the first member of a potential new genus within the *Parvovirinae* subfamily, although its existence needs first to be accepted by the advisory board of the ICTV. Still, its discovery could open new research paths aiming to search for similar viruses in other mammals including humans. The presence of high viral variability may also increase the chance that further mutations could favor a viral transmission to other species. *Artibeus jamaicensis*, a bat from the endemic family of New World leaf-nosed bats (Phyllostomidae) that only occurs in Central and South America, forages mainly on fruit and occasionally nectar, pollen and leaves [49], [50]. It regularly covers several kilometers in search of suitable food resources, thereby crossing barriers such as open water [50], [51]. This foraging behavior can bring animals in contact with humans and other animals, i.e., livestock, especially when agricultural farming areas are used as foraging grounds [39].

Beyond these newly identified bat viruses, two recent studies reported the discovery of new parvoviruses in fecal samples from insectivore bats (Rhinolophidae, Hipposideridae, Vespertilionidae, Miniopteridae and Molossidae) [12], [16]. In both studies, the detected viruses belonged to the genus *Dependovirus* that mainly includes non-autonomous viruses. Additionally, in one of these studies a virus (TM-2p) was identified which is topologically located in the phylogenetic tree between the two parvoviral subfamilies *Parvovirinae* and *Densovirinae* [12]. Since fecal samples from insectivorous bats were investigated, TM-2p might be a virus infecting insects rather than bats and gotten into stools after having been ingested through the diet [12]. Due to difficulties in alignment (only 210 aa of the less conserved part of NS1 protein of TM-2p virus were available) it was however not possible to compare this virus with our sequences.

The two parvoviruses reported here were identified from blood samples, indicating that these viruses cause active infections in bats. Beyond the 2 parvoviruses, no other viral sequences were identified during our study, suggesting that both viruses may replicate autonomously and may not need other viruses to reach high titers (up to 1.47E+10 copies/ml). Our data also indicate that the viruses can spread easily throughout the body and that they circulate within the bat population with frequencies up to 8%. Furthermore, the presence of bat parvovirus in the intestinal tissue suggests that those viruses might be present in feces, as already reported for adeno-associated bat parvoviruses. Taken together, the data from the present and previous studies [12], [16] demonstrate that a variety of parvoviruses is currently associated with bats with different geographic origin (Old and New World), different phylogenies (Pteropodidae, Phyllostomidae, Vespertilionidae, Rhinolophidae, Hipposideridae, Miniopteridae and Molossidae), different ecologies (fruit eaters and insectivores) as well as climatic ranges (temperate to tropical regions). It is highly plausible that more parvoviruses exsist in bats and identification requires extensive studies covering a large variety of bat species.
